# Development of a predictive model for assessing the risk factors associated with recurrence following surgical treatment of chronic subdural hematoma

**DOI:** 10.3389/fsurg.2024.1429128

**Published:** 2024-10-25

**Authors:** Min Chen, Longbiao Da, Qingchao Zhang, Jie Liu, Jian Tang, Zhengjiang Zha

**Affiliations:** Department of Neurosurgery, Anqing Municipal Hospital, Anqing, China

**Keywords:** chronic subdural hematoma, recurrence, statins, a predictive model, independent risk factors

## Abstract

**Background:**

Chronic subdural hematoma (CSDH) is a common disease in neurosurgery. Although many studies have investigated the factors affecting the recurrence of CSDH, no comprehensive prediction model has been established for the risk effect of postoperative recurrence of the disease.

**Objective:**

This study aims to collect and analyze the data of CSDH patients treated in our hospital to determine the influence of preoperative, postoperative and treatment factors on the recurrence of CSDH, and to establish a corresponding prediction model to provide neurosurgeons with more accurate basis for identifying high-risk patients and guiding treatment.

**Methods:**

A total of 431 patients were collected in this study, including 323 patients who underwent traditional hematoma removal and 108 patients who underwent endoscopic hematoma removal. Relevant preoperative and postoperative data and medical history of patients were collected respectively to study the relevant factors affecting postoperative hematoma recurrence of patients, and to establish a prediction model.

**Results:**

A total of 431 patients were enrolled in this study, 71 of whom had subdural blood recurrence. Possible relevant factors were included in univariate logistic regression, and the results showed that the preoperative GCS score, postoperative residual gas, preoperative CT hematoma thickness, coagulation function, unilateral and bilateral surgery, whether statin was taken after surgery, hematoma site, hematoma density and hematoma volume were all *P* < 0.2. It is a risk factor for recurrence of chronic subdural hematoma. The obtained data were further included in a multi-factor review. Six factors, including preoperative GCS score, postoperative gas residual, abnormal coagulation function, high-density hematoma, large hematoma volume, and irregular statin use after surgery, were independent risk factors for chronic subdural hematoma recurrence (*P* < 0.05).

**Conclusion:**

This study confirmed that six factors, including preoperative GCS score, postoperative gas residual, abnormal coagulation function, high-density hematoma, large hematoma volume, and irregular statin use, were independent risk factors for recurrence of chronic subdural hematoma. At the same time, long-term use of statins can reduce the recurrence rate of hematoma to a certain extent. In addition, the predictive model in this study could help neurosurgeons accurately identify high-risk CSDH patients.

## 1 Introduction

Chronic subdural hematoma (CSDH) is a common neurosurgical disease (1), which is usually caused by head trauma, or in the absence of obvious trauma, due to abnormal blood clotting or vascular lesions and other factors. The disease is characterized by a continuous accumulation of blood under the dural membrane, which gradually forms a hematoma. Because of its potentially serious consequences, such as increased intracranial pressure, brain tissue compression and dysfunction, surgical intervention is often needed to treat it. However, postoperative recurrence has always been a clinical problem plaguing medical personnel, and its recurrence rate has not been effectively controlled in the international scope (2, 3). Although numerous studies have been devoted to identifying and analyzing risk factors for postoperative recurrence of CSDH, there is still a lack of a reliable, uniform predictive model to guide clinical practice. In the world, the research on postoperative recurrence of CSDH shows a diversified trend. Some studies have focused on biological factors, such as thrombosis, angiogenesis, and inflammatory response, to explore their association with postoperative recurrence (4–6). At the same time, other studies focused on clinical factors, such as age, gender, medical history, etc., trying to establish a prediction model for postoperative recurrence (3, 7, 8). In addition, advances in imaging technology also provide new ideas and methods for the prediction of postoperative recurrence (9, 10). However, although there have been some international research results, in practice, the prediction of postoperative recurrence of CSDH still faces challenges. In China, there are relatively few relevant studies, and no systematic research framework has been formed. Therefore, it is of great clinical significance to establish a reliable postoperative recurrence prediction model suitable for Chinese patients. By collecting a large amount of clinical data and using predictive modeling methods, we will establish a prediction model for the risk impact of postoperative recurrence of CSDH in Chinese patients. The aim of our study is to provide an effective tool to help neurosurgeons accurately identify high-risk patients with CSDH and take timely personalized treatment measures to reduce the incidence of postoperative recurrence and improve patient prognosis and quality of life. At the same time, we also hope to provide new ideas and methods for further research in this field.

## 2 Materials and methods

### 2.1 Subjects

The subjects of this study were 431 patients who were hospitalized and underwent surgery in the Department of Neurosurgery of our hospital from January 2021 to June 2023, including 350 males and 81 females. The age ranged from 11 to 96 years old, with an average age of 69.16 ± 9.82. The preoperative Glasgow coma scale (GCS) score was (12.61 ± 1.74). This study was reported for approval by the ethics committee of our hospital.

### 2.2 Methods

A total of 431 patients were collected in this study, including 323 patients who underwent traditional hematoma evacuation and 108 patients who underwent endoscopic hematoma evacuation. Relevant preoperative and postoperative data and medical history were collected, and the related factors affecting postoperative hematoma recurrence were analyzed.

### 2.3 Collected indicators

This study collected the gender, age, surgical method, medical history and preoperative and postoperative related indicators of patients. Among them, preoperative indicators include preoperative hematoma thickness, preoperative hematoma separation, hematoma site, hematoma volume and hematoma density. Postoperative indicators included residual gas, catheter placement time, and statin and anticoagulant drug use.

### 2.4 Statistical methods

SPSS 25.0 was used for data processing and statistical analysis in this study. Quantitative data conforming to normal distribution were expressed as mean ± standard deviation, and the differences between groups were analyzed by independent sample T test. Comparisons between groups that did not follow a normal distribution were performed using nonparametric tests. Data for qualitative data were expressed as number of cases and percentage, and chi-square tests were used to determine whether there were differences between groups. Firstly, the recurrence of hematoma and various clinical related indicators were analyzed, and then the potential risk factors of chronic subdural hematoma recurrence were determined based on univariate logistic regression analysis of the collected data. In the univariate analysis, exposure factors with *P* ≤ 0.2 were selected and included in the multivariate analysis. The independent risk factors of chronic subdural hematoma recurrence were obtained. *P* < 0.05 was considered statistically significant.

## 3 Results

### 3.1 The assignment table of relevant indicators in this study

See Table 1 for details.

**Table 1:** Assignment table.

Name	Variable assignment and description
Group	Relapse-1, No recurrence-0
Surgical methods	Traditional surgical methods-1, Endoscope-0
Preoperative CT hematoma thickness	≤25 mm-0, >25 mm-1
Cardiopulmonary function	abnormal-1, normal-0
Coagulation function	abnormal-1, normal-0
The side of the hematoma	bilateral-1, One side-0
Saline irrigation or not	Rinse off-1, Not rinse off-0
Location of hematoma	Frontoparietal region-0, Frontotemporal parietal-1, Frontotemporal, parietal and occipital regions-2
Hematoma density	High density-1,others-2
Hematoma volume	≤160 cm^3^-0, >160 cm^3^-1

### 3.2 Univariate analysis

A total of 431 patients were enrolled in this study, 71 of whom had subdural blood recurrence. Possible relevant factors were included in univariate logistic regression, including age, preoperative GCS score, catheter placement time, postoperative residual gas, surgical method, coagulation function, intraoperative saline irrigation, and postoperative statin use. After analysis, the results showed that preoperative GCS score, postoperative residual gas, preoperative CT hematoma thickness, coagulation function, unilateral and bilateral surgery, whether to take statins after surgery, hematoma site, hematoma density and hematoma volume were the risk factors for the recurrence of chronic subdural hematoma with *P* < 0.2. See Table 2 for details.

**Table 2:** Univariate analysis of chronic subdural hematoma recurrence.

Variables	Beta	S.E	Z	*P*	OR (95% CI)
Age	0.00	0.01	0.04	0.965	1.00 (0.97–1.03)
Preoperative GCS	−0.27	0.08	−3.48	<.001	0.77 (0.66–0.89)
Duration of drainage tube placement (hour)	−0.05	0.06	−0.82	0.414	0.95 (0.84–1.07)
Postoperative residual gas (ml)	0.11	0.03	4.01	<.001	1.11 (1.06–1.17)
Gender					
male					1.00 (Reference)
female	0.18	0.32	0.55	0.582	1.19 (0.64–2.24)
Surgical methods					
0					1.00 (Reference)
1	0.82	0.36	2.28	0.022	2.28 (1.12–4.63)
Preoperative CT hematoma thickness					
0					1.00 (Reference)
1	0.90	0.27	3.36	<.001	2.46 (1.46–4.16)
The presence or absence of a partition in the preoperative CT hematoma					
0					1.00 (Reference)
1	0.50	0.26	1.92	0.055	1.65 (0.99–2.75)
Cardiopulmonary function					
0					1.00 (Reference)
1	−0.36	0.32	−1.13	0.258	0.70 (0.37–1.31)
Hypertension					
0					1.00 (Reference)
1	0.02	0.27	0.09	0.931	1.02 (0.61–1.72)
Diabetes					
0					1.00 (Reference)
1	0.19	0.27	0.68	0.498	1.20 (0.70–2.06)
Coagulation function					
0					1.00 (Reference)
1	0.97	0.28	3.45	<.001	2.63 (1.52–4.56)
Unilateral or bilateral surgery					
0					1.00 (Reference)
1	0.87	0.27	3.29	0.001	2.39 (1.42–4.02)
Intraoperative saline irrigation or not					
0					1.00 (Reference)
1	−0.22	0.26	−0.85	0.397	0.80 (0.48–1.34)
Whether to take statins after surgery					
0					1.00 (Reference)
1	−1.44	0.28	−5.10	<.001	0.24 (0.14–0.41)
Postoperative use of anticoagulant drugs					
0					1.00 (Reference)
1	0.59	0.27	2.20	0.027	1.80 (1.07–3.04)
History of cancer					
0					1.00 (Reference)
1	0.26	0.48	0.54	0.590	1.29 (0.51–3.29)
Location of hematoma					
0					1.00 (Reference)
1	1.26	0.43	2.91	0.004	3.53 (1.51–8.26)
2	0.71	0.45	1.58	0.115	2.03 (0.84–4.90)
Hematoma density					
0					1.00 (Reference)
1	−1.89	0.34	−5.47	<.001	0.15 (0.08–0.30)
Hematoma volume					
0					1.00 (Reference)
1	1.22	0.32	3.74	<.001	3.37 (1.78–6.38)

### 3.3 Multifactor analysis

The nine risk factors obtained from the univariate analysis were further incorporated into the multivariate analysis, and the results showed that preoperative GCS score, postoperative gas residual, abnormal coagulation function, high-density hematoma, large hematoma volume, and irregular postoperative statin use were independent risk factors for the recurrence of chronic subdural hematoma. See Table 3 for details.

**Table 3:** Multivariate analysis of chronic subdural hematoma recurrence.

Variables	Beta	S.E	*Z*	*P*	OR (95% CI)
Preoperative GCS	−0.29	0.11	−2.68	0.007	0.75 (0.61–0.93)
Postoperative residual gas (ml)	0.12	0.04	3.23	0.001	1.12 (1.05–1.20)
Endoscopic surgery	−0.83	0.48	−1.75	0.080	0.44 (0.17–1.11)
Abnormal coagulation function	3.18	0.53	6.03	<.001	24.01 (8.55–67.47)
Take statins after surgery	−1.21	0.37	−3.27	0.001	0.30 (0.14–0.62)
High density hematoma	3.70	0.53	7.01	<.001	40.65 (14.42–114.54)
Hematoma volume ≤160 cm^3^	−1.27	0.43	−2.95	0.003	0.28 (0.12–0.65)

### 3.4 Drawing of the nomogram

**Figure 1:**
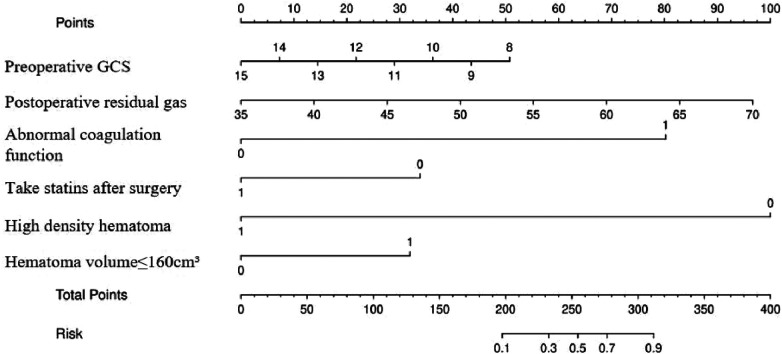
Nomogram predicting risk of chronic subdural hematoma recurred.

**Figure 2:**
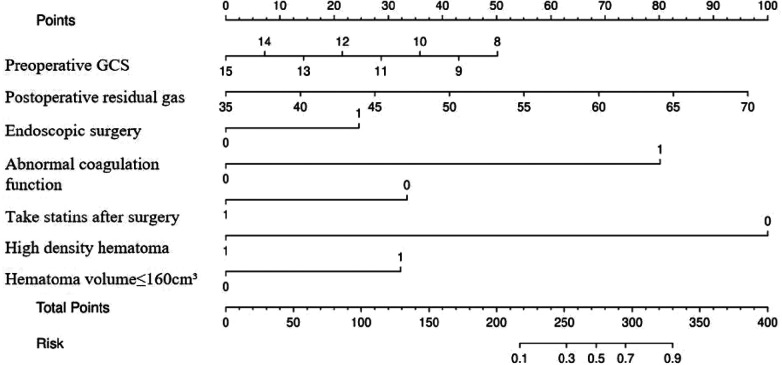
Nomogram predicting risk of chronic subdural hematoma recurred.

**Figure 3:**
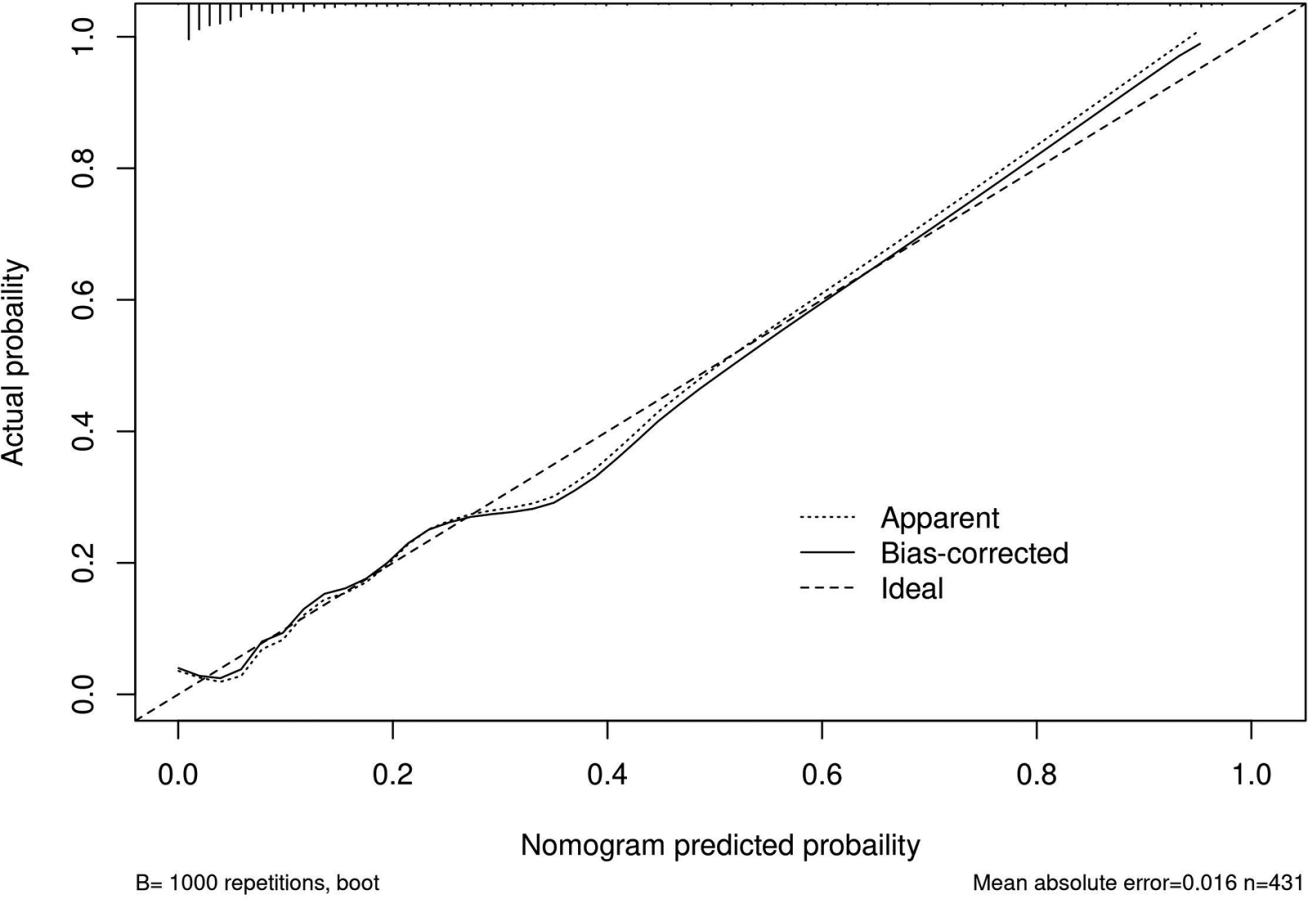
Internal validation of the column diagram: calibration curve.

**Figure 4:**
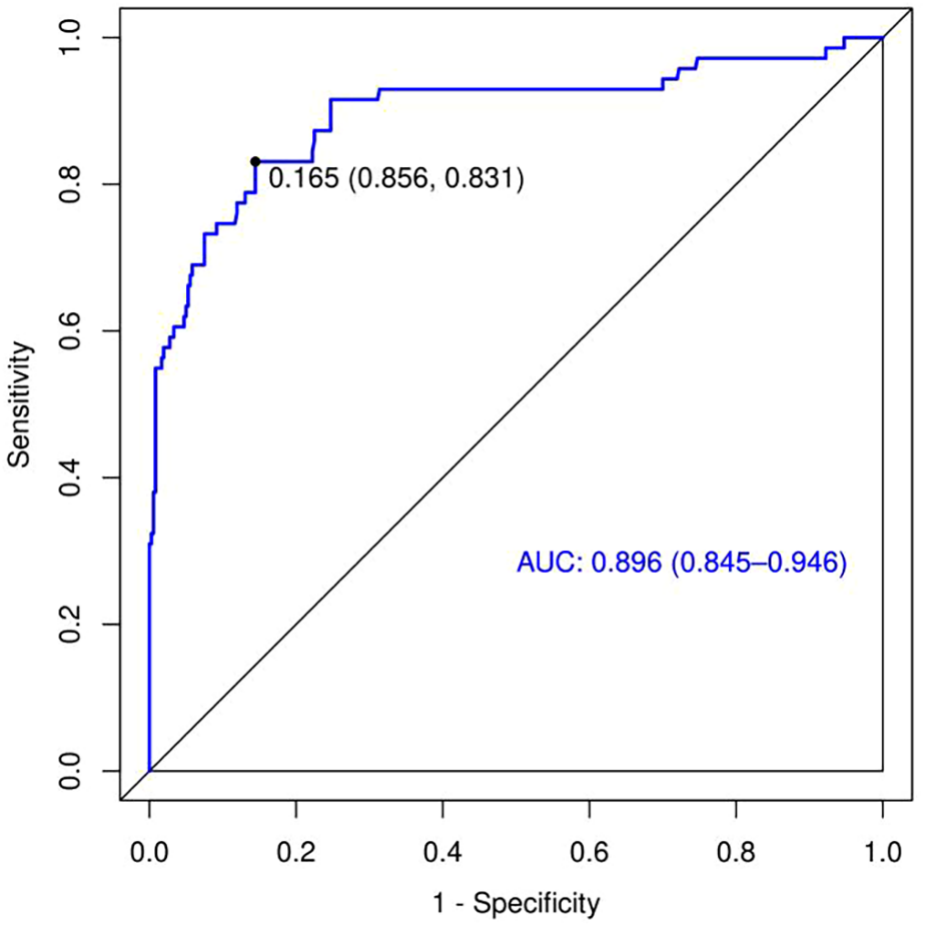
ROC curve.

**Figure 5:**
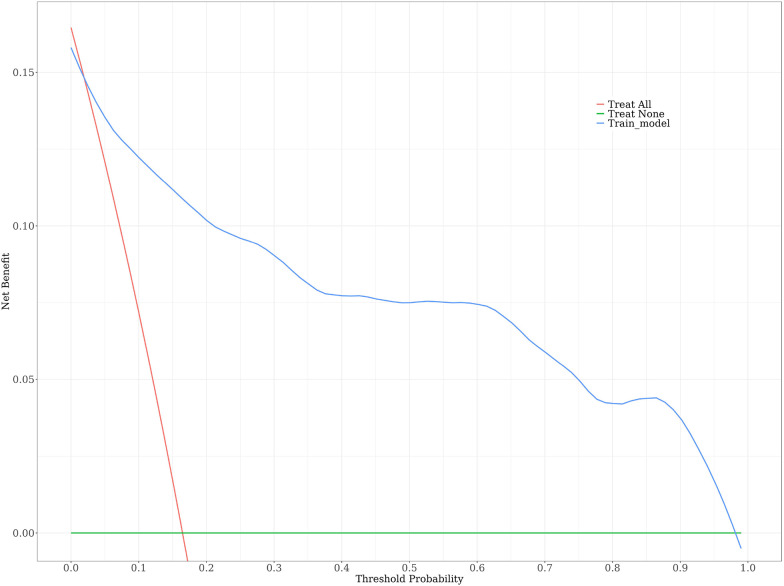
Decision curves in nomogram models.

A risk nomogram for chronic subdural hematoma recurrence was constructed based on the six independent predictors tested by multivariate logistic regression analysis, as shown in Figure 1. In addition, we added a nomogram regarding surgical modality for constructing the risk of chronic subdural hematoma recurrence, see Figure 2. The Nomo score of each independent Risk factor was assigned, and the Total score was obtained based on the sum of the clinical characteristics of the patient. Located on the Total points axis, the value on the risk axis corresponding to the vertical downward is the recurrence probability of chronic subdural hematoma of the patient, and the score of each independent predictor corresponds to the upper limit of the score of each independent predictor. The total score for each subject is the sum of the scores of the independent predictors. The risk of chronic subdural hematoma recurrence was determined by the total score value on the chronic subdural hematoma recurrence risk axis. Subsequently, the model was internally validated, and the Bootstrap method in R software was used to verify the nomogram by repeated sampling for 1,000 times. The calibration curve was close to the ideal curve, indicating that the nomogram had a high degree of agreement between the predicted incidence of chronic subdural hematoma recurrence and the actual incidence, reflecting a good predictive performance, as shown in Figure 3. The ROC curve of the nomogram training set showed an AUC of 0.896 (95% CI = 0.845–0.946, Sensitivity: 85.6%, Specificity: 83.1%), as shown in Figure 4. It indicates that the nomogram has a good discrimination degree for the high-risk population of chronic subdural hematoma recurrence. The decision curve (DCA) of the nomogram showed that when the threshold probability of an individual in the nomogram was greater than 0.05, the model provided more net benefits than the “all intervene” or “all do not intervene” strategies, which indicated the clinical utility of the nomogram model in predicting the recurrence of chronic subdural hematoma (see Figure 5).

## 4 Discussion

Chronic Subdural Hematoma (CSDH) is a common neurosurgical disorder characterized by hematoma that forms between the dura and pia meninges. The formation of CSDH is usually caused by head trauma, brain contusion, vascular disease, or hemorrhagic disease.

In this study, a total of 431 patients with chronic subdural hematoma treated in our hospital in the past few years were collected, of which 71 patients with different surgical methods relapsed (accounting for 16.47%). Multivariate regression results showed that preoperative GCS score, postoperative gas residual, abnormal coagulation function, high-density hematoma, large hematoma volume, and irregular statin use were independent risk factors for recurrence of chronic subdural hematoma.

Existing studies have found that there are many factors in the literature that affect the recurrence of chronic subdural hematoma, including the age of patients, hypertension, hematoma-related indicators, and the different treatment methods of patients (11, 12). According to the conclusions drawn in this paper, preoperative GCS score, postoperative gas residual, abnormal coagulation function, high-density hematoma, large hematoma volume, and irregular statin use after surgery are all independent risk factors for recurrence of chronic subdural hematoma, which is generally consistent with the conclusions drawn by previous scholars.

Preoperative GCS score and large hematoma volume indicate that the degree of subdural hematoma is more serious, the volume of hematoma is large, and the difficulty of brain reexpansion is increased, which is easy to lead to residual effusion after operation, and then increase the risk of recurrence. High density hematoma is also an independent risk factor for the recurrence of chronic subdural hematoma. The authors of this study analyzed that the hematoma density represents the proportion of new blood clots in the hematoma, and the higher the density often represents the higher the proportion of new blood clots and the more severe the fresh bleeding. Existing studies have pointed out that the progression of chronic submeningeal hematoma is often divided into three stages, namely the early stage (fibrinolysis and coagulation are relatively balanced), the middle stage (fibrinolysis is enhanced, the hematoma is differentiated into low density and high density, and the hematoma volume increases, leading to brain tissue pressure and increased bleeding risk), and the late stage (bleeding tendency decreases). The middle stage patients have a higher risk of rebleeding. It also increases the probability of hematoma recurrence (11).

The benefit of statins in reducing the recurrence rate of hematoma has been confirmed by other clinical studies. Some studies have shown that statins can significantly reduce the body's cholesterol level by competitively inhibiting the endogenous cholesterol synthesis limiting enzyme HMG-coa reductase, which plays a role in stabilizing the blood vessel wall, inhibiting angiogenesis and inflammation. This has a positive significance in reducing vascular permeability, and has a positive effect on reducing the recurrence rate of hematoma (13). According to the authors of this study, although the results of this study suggest that statins can help to reduce the recurrence rate of hematoma, long-term follow-up observation is still needed to determine whether patients with chronic submeningeal hematoma need to take statins for a long time after surgery.

It is worth noting that the conclusions of this study are based on the analysis and collation of the existing literature, and although the results are somewhat reliable, there are still some limitations. The study is conducted at a single center, this is a limitation of our study, which may limit the generalizability of the findings to other populations or healthcare settings. In the next study, we will conduct multi-center prospective studies to validate the findings and enhance the generalizability and reliability of the results of predictive model across different patient populations and clinical settings would. Meanwhile, the predictive model has not been validated with an external dataset. We will validati the predictive model with external datasets to confirm its applicability and reliability in diverse clinical scenarios. The follow-up period is short, which may not be long enough to capture all instances of recurrence, particularly late recurrences. For further research, we will extending the follow-up period to capture long-term outcomes and late recurrences to provide a more comprehensive understanding of recurrence risk. There is a notable imbalance between the number of patients undergoing traditional hematoma removal and those undergoing endoscopic hematoma removal, which could cause bias. In addition, there may be other unmeasured confounding factors that could influence the recurrence of CSDH. In future prospective studies, the number of patients receiving different surgical techniques will be more balanced to reduce bias and improve the reliability of research results.

## 5 Conclusions

Based on the results of this study, six factors including preoperative GCS score, postoperative gas residual, abnormal coagulation function, high-density hematoma, large hematoma volume, and irregular use of statins after surgery were confirmed as independent risk factors for the recurrence of chronic subdural hematoma. In addition, the long-term use of statins helps to reduce the recurrence rate of hematoma to a certain extent. In addition, the prediction model developed in this study can help neurosurgeons to accurately identify high-risk CSDH patients.

## Data Availability

The raw data supporting the conclusions of this article will be made available by the authors, without undue reservation.
